# Effect of a flipped classroom course to foster medical students’ AI literacy with a focus on medical imaging: a single group pre-and post-test study

**DOI:** 10.1186/s12909-022-03866-x

**Published:** 2022-11-18

**Authors:** Matthias C. Laupichler, Dariusch R. Hadizadeh, Maximilian W. M. Wintergerst, Leon von der Emde, Daniel Paech, Elizabeth A. Dick, Tobias Raupach

**Affiliations:** 1 Institute of Medical Education, University Hospital Bonn, Venusberg-Campus 1, 53127 Bonn, Germany; 2 Clinic for Diagnostic and Interventional Radiology, University Hospital Bonn, Bonn, Germany; 3 Department of Ophthalmology, University Hospital Bonn, Bonn, Germany; 4 Clinic for Neuroradiology, University Hospital Bonn, Bonn, Germany; 5 Imperial College NHS Trust and Imperial College London, St. Marys Hospital London, London, UK

**Keywords:** AI readiness, AI literacy, Artificial intelligence, Flipped classroom

## Abstract

**Background:**

The use of artificial intelligence applications in medicine is becoming increasingly common. At the same time, however, there are few initiatives to teach this important and timely topic to medical students. One reason for this is the predetermined medical curriculum, which leaves very little room for new topics that were not included before. We present a flipped classroom course designed to give undergraduate medical students an elaborated first impression of AI and to increase their “AI readiness”.

**Methods:**

The course was tested and evaluated at Bonn Medical School in Germany with medical students in semester three or higher and consisted of a mixture of online self-study units and online classroom lessons. While the online content provided the theoretical underpinnings and demonstrated different perspectives on AI in medical imaging, the classroom sessions offered deeper insight into how “human” diagnostic decision-making differs from AI diagnoses. This was achieved through interactive exercises in which students first diagnosed medical image data themselves and then compared their results with the AI diagnoses. We adapted the “Medical Artificial Intelligence Scale for Medical Students” to evaluate differences in “AI readiness” before and after taking part in the course. These differences were measured by calculating the so called “comparative self-assessment gain” (CSA gain) which enables a valid and reliable representation of changes in behaviour, attitudes, or knowledge.

**Results:**

We found a statistically significant increase in perceived AI readiness. While values of CSA gain were different across items and factors, the overall CSA gain regarding AI readiness was satisfactory.

**Conclusion:**

Attending a course developed to increase knowledge about AI in medical imaging can increase self-perceived AI readiness in medical students.

**Supplementary Information:**

The online version contains supplementary material available at 10.1186/s12909-022-03866-x.

## Background

### Artificial intelligence education in medical schools

Developments of artificial intelligence (AI) in healthcare accelerated in the past few years. Already today, a number of medical fields rely on the help of AI-programs in their work [1, 2], including radiology [3], cardiology [4], or drug design [5], albeit mostly in laboratory or experimental environments. While AI and its currently most important technology, machine learning, penetrates more and more medical subspecialties, it is most prevalent in medical imaging [6–10]. There is a clear positive trend in the funding of AI research in the healthcare sector. This applies both to the private and venture capital sector, which has seen a steady increase in funding over the last few quarters [11, 12], as well as to the public sector. The governments of several countries are promoting the research and implementation of AI in healthcare, such as the USA with the Department of Health & Human Services’ “Artificial Intelligence Strategy” [13], Germany as part of their “National Strategy for Artificial Intelligence” [14], and China through its National Natural Science Foundation of China [15]. It can be assumed that these research results of these initiatives will reach clinical practice in the not too distant future.

However, the current medical school curricula very rarely cover aspects of medical informatics, let alone AI, as part of their mandatory study programs [16–18]. While there are some attempts to teach AI to (future) health care providers [19, 20] and to foster “AI literacy”, a term coined by Duri Long and Brian Magerko [21], the educational landscape is very heterogeneous. In addition to this, the medical curriculum in Germany and in other countries is strongly regulated by the government, listing in detail which topics must be taught during the 6 years of studying medicine (National Competence Based Learning Objectives Catalog, NKLM, www.nklm.de/zend). These relatively strict guidelines lead to little room to integrate courses about AI in regular teaching. This means that only a small fraction of medical students gets the opportunity to further their education in this important topic, although past research has already demonstrated that medical students would like to see more elaborate courses on digital health and AI [22, 23].

Apart from the fact that AI in medicine is an exciting and fast-moving research field in which future physicians and physician-scientists can conduct interesting experiments, knowing how it works and being able to interpret its results is also a prerequisite for their future jobs. Furthermore, AI will permeate much of the daily clinical work as a supporting entity [24], which will have an enormous impact on skills necessary for future practice [25]. However, medical students today do not feel they understand the subject matter particularly well or receive sufficient teaching on the topic [26].

It is important to note that promoting AI skills in medical education and increasing medical students AI readiness does not necessarily mean that all students must learn to program or understand the mathematical models underlying AI. Rather, training in AI for students from diverse study backgrounds who are not majoring in technical subjects should be about understanding how different AI applications work and what opportunities or threats come with the usage of these new technologies. As Long et al. nicely put it, the goal is to acquire a “casual understanding of AI - i.e., understanding how a search engine works, not necessarily understanding how to program one” [27].

### Creation of a flipped classroom AI course for medical students

To address the aforementioned issue, the Institute of Medical Education at Bonn Medical School, together with experts from the department of radiology, ophthalmology, and neuroradiology, created the course “KI-LAURA” (the German abbreviation stands for “Künstliche Intelligenz in der Lehre der Augenheilkunde und Radiologie” and translates to “Artificial intelligence in the teaching of ophthalmology and radiology”).

KI-LAURA was a seven-month project which was subdivided into two sections. The first part dealt with the production of online self-study content, which was subsequently uploaded to the publicly accessible MOOC platform “AI-Campus” (or “KI-Campus” in German, www.ki-campus.org). In the second section of the project, a flipped classroom course was created at Bonn campus, which consisted of the on-demand online content on the one hand and interactive classroom sessions (held online due to Covid-19 restrictions) on the other.

In addition to explanations on how AI is (and will be) supporting the diagnostic process using medical image data, possible opportunities and risks of AI application and the future of physicians in the context of AI were discussed.

The flipped classroom incorporated the online content as one component, supplemented it with more in-depth explanations, and tutored exercises (see the supplementary material for a detailed description of the course curriculum). A description of the radiology classroom session, which was held by two experts in the field of AI in radiology, can be used as an example for the interactive classroom sessions: At the beginning of the lesson, a short input on AI and medical imaging was given by a clinical expert. This was followed by a diagnostic exercise using a web-based DICOM viewer (see get.pacsbin.com), which allowed students to view medical imaging data (e.g., CT-data sets) of real, anonymized patient data on their mobile devices or PCs. Finally, an AI researcher explained how AI-algorithms are able to analyze images, and presented studies in which AI-applications are already on the level of a human radiologist in terms of diagnostic accuracy.

### Advantages of using the flipped classroom method for AI education in medical schools

Using the flipped classroom method to develop students’ AI competencies has several advantages, which are even more important in the context of medical education. First, knowledge transfer components are separated in time and space from in-depth practical teaching [28], which increases perceived learning flexibility [29] and student satisfaction [30]. Second, due to the low implementation effort, other medical schools can easily adopt the course structure, which means that the course is scalable with little to no additional resource requirements. Third, the increased flexibility and reduced effort ensures that the course can be easily integrated into existing medical curricula without the need for major changes. Last but not least, this kind of teaching format seems to be particularly suitable in times of the Covid-19 pandemic [31], as it makes it possible to switch spontaneously between online and face-to-face teaching since the online content is already available in its entirety.

### Measuring changes in AI readiness

Karaca et al. [32] recently created the so-called “Medical Artificial Intelligence Readiness Scale for Medical Students” (MAIRS-MS), which tries to assess the perceived preparedness to use AI-applications in healthcare, i.e., “AI readiness”. The scale was psychometrically tested for reliability and validity on a population of Turkish medical students and achieved good results on both criteria. However, that scale was not originally designed to detect changes in AI readiness, but rather to evaluate the status quo of the concept. Nevertheless, it makes sense to resort to this scale and adapt it as an instrument to measure changes in AI readiness, as it is the only available instrument that has been psychometrically tested and developed specifically for medical students.

### Purpose of the research

The main purpose of this study was to design and evaluate a novel AI-course for medical students. In addition to students’ attitudes towards the course, our primary research objective was to assess if and in what ways the course changed the AI readiness of the participants. Thus, this research attempts to answer two questions:

1. What are students’ attitudes towards the course on AI in medical imaging?
2. Does the AI-course presented here have an effect on students’ perceived AI readiness?

## Methods

### Setting and participants

We conducted our research at Bonn Medical School in Germany in the winter semester of 2021/22. Medical students from semester three or higher were invited via messenger apps to participate in the aforementioned voluntary, extracurricular course about AI in medical imaging. While they could not obtain credit points or grades, they were incentivized by the opportunity to receive a certificate of extracurricular achievement if they attended the individual lessons and passed four multiple choice tests (graded learning progress tests).

As mentioned before, due to restrictions caused by the Covid-19 pandemic, “classroom” lessons were held through an online conference tool. All self-study content could be accessed and downloaded by students free of charge and for an unlimited time on the MOOC platform “AI-Campus” after an initial registration on the website.

### Response rate and participants’ characteristics

From an initial 32 course participants, 3 dropped out during the course and 5 did not respond to both questionnaires, leading to an overall response rate of 83% and a final sample of *n* = 24.

There were 21 women (87.5%) and 3 men (12.5%). The median semester that participants reported was 7 (min = 5, max = 11). Participants’ age ranged from 21 to 37 years, with a median of 23 (see Tables 1 and 2).

**Table 1:** Participants’ gender

Gender	Absolute	Relative
Male	3	12.5%
Female	21	87.5%

**Table 2:** Participants’ semester and age

	Mean	Median	SD	Min	Max
Semester	7.2	7	2.12	5	11
Age	24.0	23	3.96	21	37

### Data collection

Data collection was conducted prior to each of the last two lessons. Online questionnaires were distributed to the course participants via link sharing. We informed students that study participation was voluntary. The first questionnaire consisted of an adaptation of the “Medical Artificial Intelligence Readiness Scale for Medical Students (MAIRS-MS) [32] and the second asked for more general assessments (see “General evaluation” below). In order to simultaneously preserve anonymity and enable comparability between the two measurements, a subject code was generated at the beginning of the first questionnaire, which was queried again in the second questionnaire. The completion of each questionnaire took less than 10 minutes.

#### MAIRS-MS

The original MAIRS-MS instrument asks participants to rate their self-assessment on a 5-point Likert-scale (ranging from 1 - “strongly disagree” to 5 - “strongly agree”) on 22 statements regarding their own AI readiness. The authors define AI readiness as “the healthcare provider’s preparedness state in knowledge, skills, and attitude to utilize healthcare-AI applications during delivering prevention, diagnosis, treatment, and rehabilitation services in amalgam with own professional knowledge” [30]. MAIRS-MS is subdivided into four factors: “cognition” (8 items), “ability” (8 items), “vision” (3 items), and “ethics” (3 items). Two exemplary items are called “I can explain how AI systems are trained” (cognition) and “I can explain the limitations of AI technology” (vision) (see Fig. 2 for an exhaustive list of all items). In the original study, the authors report the internal consistency of the whole scale to be acceptable (Cronbach’s alpha coefficient = 0.88), and Cronbach’s alpha for the individual factors was 0.83 (cognition), 0.77 (ability), 0.72 (vision), and 0.63 (ethics), respectively.

#### Adaptation of MAIRS-MS

Since the MAIRS-MS instrument was designed to assess the status-quo of AI readiness in medical students, some changes to the original questionnaire were necessary. While the general structure remained the same, each item was presented in a “post”-version (self-assessment *after* attending the course) and a “then”-version (retrospective self-assessment *before* attending the course). In addition, the questionnaire was independently translated from English into German by the first author and three colleagues. There were no major discrepancies between the individual translations.

#### General evaluation

The second questionnaire consisted of 32 short questions that asked the participants, among other questions, how they liked the respective course modules and how clearly the course was structured. Socio-demographic questions covered age, semester and gender.

### Analysis

To answer research question 1 (RQ1), we conducted a repeated measures ANOVA to compare student ratings regarding self-study content (i.e., online content), classroom lessons, and the final assignment.

Since the focus of our primary research question (RQ2) lay on the assessment of differences in AI readiness, paired t-tests were calculated to determine the statistical significance of then/post differences.

However, t-tests are only able to determine that an intervention led to a change in, for example, attitude, knowledge, or skills. The problem with only reporting t-tests is that it is relatively self-evident that some changes will occur when participating in an intervention. In order to address this problem, the “comparative self-assessment gain” (CSA gain) was also calculated [33]. This measurement accounts for the initial readiness of students and thus facilitates a valid estimation of the actual increase in AI readiness through the course. CSA gain was calculated for each item and for each factor individually. It is computed as follows:

$\textrm{Aggregated}\ \textrm{performance}\ \textrm{gain}\ \left[\%\right]=\frac{{\overline{x}}_{pre}-{\overline{x}}_{post}}{{\overline{x}}_{pre}-1}\ast 100$, with ${\overline{x}}_{pre}$ meaning initial self-assessment and ${\overline{x}}_{post}$ meaning self-assessment after the course. In order to get positive values when the ratings are higher after the teaching intervention than before, each item value is recoded so that “5” would consequently mean “1”, “4” would mean “2”, and vice versa. In theory, CSA gain can range from − 100 to + 100%.

To evaluate the psychometric quality of the instrument, the sum of each item (i.e., addition of each participant’s rating for each factor) in the factors (“AI-cognition”, “-ability”, “-vision” and “-ethics”) were correlated with each other and Cronbach’s alpha was calculated as a measurement of reliability.

We have refrained from a gender-based, group-wise mean comparison, as only 3 male participants (compared to 21 females) took part in the survey. Furthermore, we only included data of participants who answered both questionnaires, leading to the exclusion of 5 subjects who only answered one questionnaire.

We used the statistics software SPSS (see www.ibm.com/analytics/spss-statistics-software), R (with R-Studio, see www.rstudio.com), and Python 3 (see www.python.org) for the analyses and data visualizations and set significance levels to 5%. We present descriptive data as means (or medians, where appropriate) and standard deviations (*SD*s), and we present correlations as Pearson r (95% confidence interval).

## Results

### General evaluation

Twelve participants (50.0%) graded the overall course “1 - very good” (German grading system, 1 corresponds to “A” in the American grading system, 6 corresponds to “F”), 11 participants (45.8%) rated it with “2 - good” and 1 person (4.2%) gave it a “3 - satisfactory”. Furthermore, 23 participants (95.8%) stated that their motivation to participate was their interest in the topic and/or the relevance of the topic for their future profession, respectively (multiple answers were possible). Nineteen subjects also considered the possibility to obtain a certificate as being a primary motivator, and 16 thought the topic to be relevant for their further studies. In addition to that, one person also ticked “Other, namely…” and indicated in the free text field that they wanted to try out this new format of continuing education.

### Comparison between satisfaction with online self-study content, classroom lessons, and final assignment

**Fig. 1:**
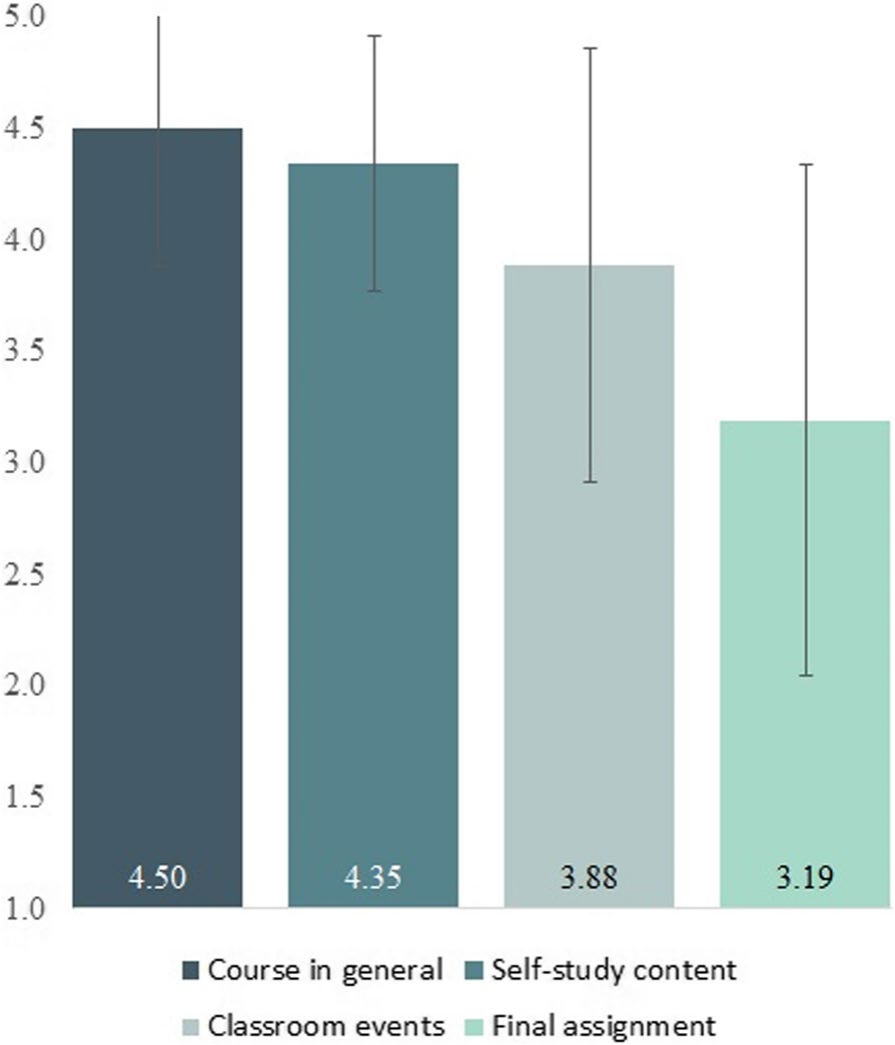
Student evaluation of formats used in the course. Students rated the course on a Likert-scale ranging from 1 to 5

The mean student rating on the course in general was 4.50 on a 5-point Likert-scale (*SD* = 0.57). The repeated measures ANOVA determined that mean student ratings showed a statistically significant difference between the three pedagogical formats (i.e., self-study content, classroom lessons, and final assignment), *F*(2, 46) = 15.33, *p* < .001, partial η^2^ = .40. While the mean rating of the self-study content was higher than the mean rating of the classroom sessions (see Fig. 1), Bonferroni-adjusted post-hoc analysis revealed this difference to be statistically insignificant (*p* = .06, M_Diff_ = 0.46, 95%-CI [− 0.01, 0.92]). However, post-hoc analysis found that both the self-study content and the classroom sessions were rated significantly more positive than the final assignment (*p* < .001, M_Diff_ = 1.25, 95%-CI [0.57, 1.93] and *p* < .01, M_Diff_ = 0.79, 95%-CI [0.19, 1.39], respectively).

### Change in perceived AI readiness

Mean self-ratings after course completion (post-items) were significantly higher than mean self-ratings before course participation (then-items) for the cognition factor, *t* (23) = 16.09, *p* < .001, the ability factor, *t* (23) = 13.41, *p* < .001, the vision factor, *t* (23) = 12.61, *p* < .001, and the ethics factor, *t* (23) = 6.85, *p* < .001, respectively.

**Fig. 2:**
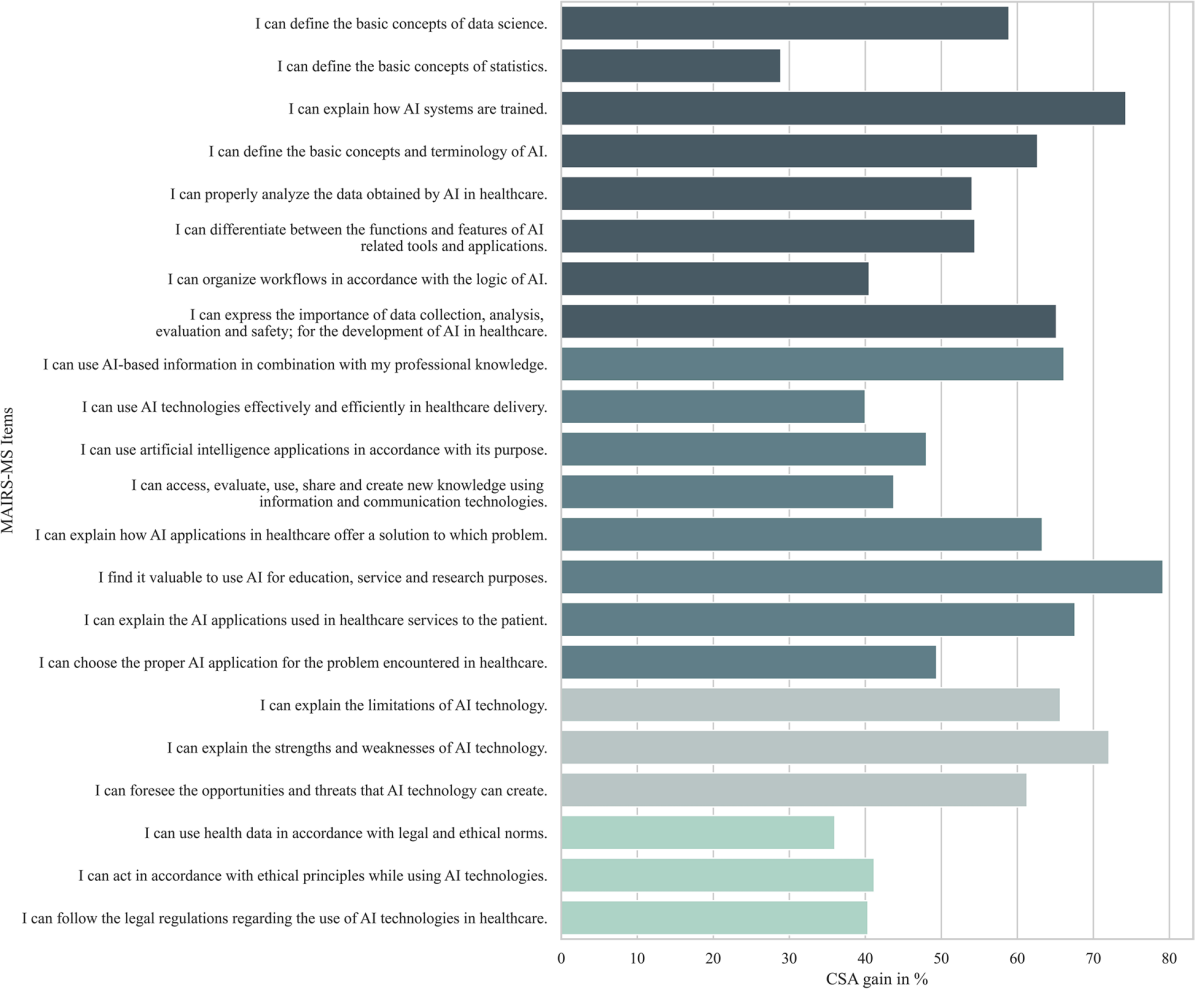
Comparative Self-Assessment Gain in percent for every MAIRS-MS item. Note: The colors indicate to which factor each item belongs. The factor order is cognition, ability, vision, and ethics

As described earlier, since t-tests are generally limited in their ability to explain or compare changes resulting from participation in an intervention (see “Analysis” section), CSA gain was also calculated. The aggregated CSA gain (CSA gain on group-level) was positive on all 22 items. While the mean CSA gain averaged across all items was 55.6% (*SD* = 12.9), which can be interpreted as an acceptable result of an intervention, CSA gain scores differed widely across individual items (see Fig. 2). The three items that generated the lowest CSA gain were “I can define the basic concepts of statistics.” (cognition-factor, 28.3%), “I can use health data in accordance with legal and ethical norms.” (ethics-factor, 37.3%), and “I can use AI technologies effectively and efficiently in healthcare delivery.” (ability-factor, 38.8%). The three items that generated the highest CSA gain were “I can explain the strengths and weaknesses of AI technology.” (vision-factor, 72.2%), “I can explain how AI systems are trained.” (cognition-factor, 72.8%), and “I find it valuable to use AI for education, service and research purposes.” (ability, 73.5%).

On a factor-level, the vision-factor yielded the biggest CSA gain (67.1%, *SD* = 13.0), followed by ability (57.3%, *SD* = 12.0), cognition (55.1%, *SD* = 4.3), and ethics (40.8%, *SD* = 2.9).

### Psychometric properties and internal consistency of the questionnaire

The internal consistency of both whole scales, measured by Cronbach’s alpha, was […].93 (“then”-condition) and .88 (“post”-condition), which can be interpreted as good [34] (see Tables 3 and 4). Cronbach’s alpha of the individual factor-scales was good or acceptable (.78 to .88) with one exception: The internal consistency of the “post”-cognition factor was only .67.

**Table 3:** Descriptive statistics and reliability of “then”-factors

“Then”- Factors	1	2	3	4	Mean	SD	Cronbach’s Alpha	Skewness	Kurtosis
1. Cognition					14.5	5.47	.88	1.24	4.04
2. Ability	.80***				15.3	4.21	.81	1.23	3.69
3. Vision	.66***	.75***			6.2	2.48	.86	0.56	2.60
4. Ethics	.35	.56**	.49*		7.6	3.28	.81	0.73	2.57

* *p* < .05, ** *p* < .01, *** *p* < .001

**Table 4:** Descriptive statistics and reliability of “post”-factors

“Post”- Factors	1	2	3	4	Mean	SD	Cronbach’s Alpha	Skewness	Kurtosis
1. Cognition					29.1	3.19	.67	−0.35	1.84
2. Ability	.49*				29.2	4.51	.78	0.87	2.90
3. Vision	.55**	.56**			12.2	1.91	.86	−0.27	2.42
4. Ethics	.28	.62**	.60**		10.7	2.71	.82	0.31	2.05

* *p* < .05, ** *p* < .01, *** *p* < .001

## Discussion

The results of our study indicate that the implementation of a flipped classroom AI-course is a viable option to teach AI to medical students and increase their AI readiness. Thus, our research tries to answer the call expressed by many scholars that AI competencies of medical students should be promoted [35] and that their AI readiness should be improved, as there is a strong student interest in this topic [36].

Students generally rated the course as “Very good” or “Good”, which provides a good indicator for student satisfaction. The marginally significant difference between self-study and face-to-face content is an indication that students might prefer the online elements to the more active classroom sessions. However, too much weight should not be placed on this difference, as the classroom lessons took place face-to-face via online video conference tools, but not in the actual classroom due to pandemic restrictions, which may have had a detrimental effect on the evaluation of the latter. Nevertheless, participants did not find the self-study content any worse than traditional teaching methods. One possible explanation for the relatively poor evaluation of the final assignment (compared to the self-study and instructional content) could be the method chosen. The course participants themselves were seen in the final assignments’ educational videos as they explained the subject matter, which may have put some participants in an unfamiliar or even intimidating situation. In future course iterations, it has to be examined whether the positive effect of active learning and independent acquisition of knowledge can also be achieved in a way that allows greater flexibility with regard to the mode of presentation.

While student satisfaction is certainly important for boosting motivation to learn and reducing dropout rates, it says little in itself about the success of the new innovation. Therefore, the adapted MAIRS-MS instrument was used to capture changes in AI readiness and to answer our main research question (RQ2). Somewhat unsurprisingly, we found statistically significant differences between self-assessments at the time before the course began and self-assessments after the course was completed.

As mentioned earlier, it is quite obvious and has been proven numerous times that attitudes, skills, etc. (e.g., AI readiness) change when participating in an intervention that is designed to improve or change these specific variables. For this reason, we decided to additionally calculate the so-called CSA gain, which takes into account the initial AI readiness of students and thus facilitates a valid estimation of the actual increase in AI readiness through the course. The CSA gain differed widely across items (see Fig. 2), with the lowest item (“I can define the basic concepts of statistics.”) at only 28.3% and the highest (“I find it valuable to use AI for education, service and research purposes.”) at 73.5%. This broad range led to an average CSA gain of 55.6%, which can just be considered “acceptable”, since good learning outcomes usually yield a gain of 60% or higher [35]. A possible explanation for the large variance between items lies in the content of the KI-LAURA course. The MAIRS-MS instrument asks very generally about AI readiness and to some extent also contains techno-mathematical aspects (see item on statistics skills), which is not considered to be a central aspect of AI literacy by all research groups (e.g., [21]). In contrast, the described course dealt with a more basic understanding of AI and focused on fundamentals (like chances and opportunities) rather than programming or mathematics.

In addition, the various modules repeatedly tried to give participants an idea of the importance of AI in the medical future, which may explain the high scores on the vision factor (67.1%). Time constraints prevented us from integrating a standalone and in-depth module discussing ethical implications, and ethical issues were only addressed in the context of higher-level problems, which explains the low CSA gain on the ethics factor (40.8%). These findings also corroborates the notion that CSA gain validly captures what was learned and what was not. Since ethics are an integral part of AI literacy education, especially in the medical context, future AI literacy courses should try to incorporate at least some basic information about the topic.

### Strengths

Our research demonstrated that the flipped classroom approach might provide a valid way to introduce medical students to AI in medical imaging. Furthermore, we found preliminary evidence that the only currently existing scale for assessing AI readiness (MAIRS-MS), which captures AI readiness at one point in time, is also able to capture intrapersonal changes in AI readiness caused by interventions.

Finally, we went beyond simply reporting mean differences and demonstrated a more valid alternative to commonly used learning effect analyses by calculating CSA gain, a tool that is much more suitable for measuring learning outcomes and changes in knowledge and skills whenever summative objective tests at the beginning as well as at the end of the course are not an option.

### Limitations

Although the psychometric qualities of the MAIRS-MS instrument were evaluated in the original study of Karaca et al. [34], we could not run a confirmatory factor analysis to check the assumption of an underlying four factor model for the adapted version of the scale due to our comparatively small sample size of 24 students. Furthermore, the original scale was created to assess AI readiness as a status quo, and the adaptation of the scale as a tool to compare AI readiness between different points of time has to be validated in future research. In addition to that, most students (95.8%) stated that they participated in the extracurricular, voluntary course because of their intrinsic interest in the topic. This could have influenced the responses in different ways. For example, it is possible that students who are interested in AI from the outset may feel more ready to use AI in their field even before taking a course, as compared to uninterested students.

Finally, MAIRS-MS tests AI readiness, a construct that describes subjects’ perceived readiness to work with AI in a professional setting. However, it would be of great interest to additionally be able to reliably measure the more commonly used “AI literacy”, as this construct deals with knowledge and skills (instead of attitudes and affect). This could be achieved by using performance tests (e.g., knowledge tests) rather than self-assessment questionnaires.

## Conclusion

AI skills training for medical students is becoming increasingly relevant. For this reason, researchers and teachers from Bonn Medical School have developed the AI course “KI-LAURA” which aims to improve medical students’ AI readiness in a flipped classroom format. To test the effectiveness of the course, the “MAIRS-MS” instrument by Karaca et al. was used. The results showed that the course significantly improved participants’ AI readiness, with a particularly high increase in AI vision. This illustrates that even entry-level AI courses can increase medical students’ AI readiness, preparing them to collaborate with AI in their future careers.

## Supplementary Information

**Additional file 1: Supplementary Table 1.** Course curriculum, subdivided into an introduction to AI, three main modules, and the final assignment.

**Additional file 2: Supplementary Table 1.** Dataset of MAIRS-MS questionnaire with “then-” and “post-” items. Note: Item VP01_01 is a pseudonym generated to enable comparisons between the self-assessment of AI readiness and the evaluation results of the blended learning course. All items ending in “01” (e.g., “CO10_01”) represent ratings after attending the course, while all items ending in “02” (e.g., “CO10_02”) reflect retrospective ratings before attending the course. **Supplementary Table 2.** Dataset of evaluation results. Note: Item VP01_01 is a pseudonym generated to enable comparisons between the self-assessment of AI readiness and the evaluation results of the blended learning course.

**Additional file 3.** Adapted Version of MAIRS-MS Questionnaire (English version).

**Additional file 4.** Adapted Version of MAIRS-MS Questionnaire (German version).

## Data Availability

The anonymized datasets used and analysed during the current study are provided as online supplementary material.

## Abbreviations

AI: Artificial Intelligence
CSA gain: Comparative Self-Assessment Gain
MAIRS-MS: Medical Artificial Intelligence Readiness Scale for Medical Students
MOOC: Massive Open Online Course
NKLM: National Competence Based Learning Objectives Catalog (German: Nationaler Kompetenzbasierter Lernzielkatalog Medizin)
RQ: Research question

## References

[1] Topol EJ (2019). High-performance medicine: the convergence of human and artificial intelligence. Nat Med.

[2] Secinaro S, Calandra D, Secinaro A, Muthurangu V, Biancone P (2021). The role of artificial intelligence in healthcare: a structured literature review. BMC Med Inform Decis Mak.

[3] Hosny A, Parmar C, Quackenbush J, Schwartz LH, Aerts HJWL (2018). Artificial intelligence in radiology. Nat Rev Cancer.

[4] Johnson KW, Torres Soto J, Glicksberg BS, Shameer K, Miotto R, Ali M (2018). Artificial Intelligence in Cardiology. J Am Coll Cardiol.

[5] Hessler G, Baringhaus KH (2018). Artificial intelligence in drug design. Molecules..

[6] Dalmış MU, Vreemann S, Kooi T, Mann RM, Karssemeijer N, Gubern-Mérida A (2018). Fully automated detection of breast cancer in screening MRI using convolutional neural networks. J Med Imaging.

[7] Bickelhaupt S, Jaeger PF, Laun FB, Lederer W, Daniel H, Kuder TA (2018). Radiomics based on adapted diffusion kurtosis imaging helps to clarify Most mammographic findings suspicious for Cancer. Radiology..

[8] Nagel S, Sinha D, Day D, Reith W, Chapot R, Papanagiotou P (2017). E-ASPECTS software is non-inferior to neuroradiologists in applying the ASPECT score to computed tomography scans of acute ischemic stroke patients. Int J Stroke.

[9] Langerhuizen DWG, Janssen SJ, Mallee WH, van den Bekerom MPJ, Ring D, Kerkhoffs GMMJ (2019). What are the applications and limitations of artificial intelligence for fracture detection and classification in Orthopaedic trauma imaging? A systematic review. Clin Orthop Relat Res.

[10] Wintergerst MWM, Jansen LG, Holz FG, Finger RP (2020). Smartphone-Based Fundus Imaging–Where Are We Now?. Asia Pac J Ophthalmol (Phila).

[11] CB Insights Research (2020). State Of Healthcare Q2 2020 Report: Sector & Industry Investment Trends | CB Insights Research. [online] CB Insights Research.

[12] CB Insights Research, 2020b. State Of Healthcare Q3 2020 Report: Sector & Industry Investment Trends | CB Insights Research. [online] CB Insights Research. Available at: https://www.cbinsights.com/research/report/healthcare-trends-q3-2020 [Accessed 24 Aug 2022].

[13] Office of the Chief Information Officer, 2021. HHS Artificial Intelligence (AI) Strategy. [online] HHS.gov. Available at: https://www.hhs.gov/about/agencies/asa/ocio/ai/strategy/index.html [Accessed 24 Aug 2022].

[14] BMBF Referat für Künstliche Intelligenz, 2022. Nationale Strategie für Künstliche Intelligenz. [online] Ki-strategie-deutschland.de. Available at: https://www.ki-strategie-deutschland.de/home.html [Accessed 24 Aug 2022].

[15] O'Meara, S., 2021. China’s data-driven dream to overhaul health care. [online] Nature.com. Available at: https://www.nature.com/articles/d41586-021-02694-1 [Accessed 24 Aug 2022]. 10.1038/d41586-021-02694-1 34616079

[16] Sit C, Srinivasan R, Amlani A, Muthuswamy K, Azam A, Monzon L (2020). Attitudes and perceptions of UK medical students towards artificial intelligence and radiology: a multicentre survey. Insights Imaging.

[17] Mosch L, Back DA, Balzer F, Bernd M, Brandt J, Erkens S, et al. Lernangebote zu Künstlicher Intelligenz in der Medizin. Berlin: KI-Campus. 10.5281/zenodo.5497668.

[18] Grunhut J, Wyatt AT, Marques O (2021). Educating future physicians in artificial intelligence (AI): an integrative review and proposed changes. J Med Educ Curric Dev.

[19] Aulenkamp J, Mikuteit M, Löffler T, Schmidt J (2021). Overview of digital health teaching courses in medical education in Germany in 2020. GMS J Med Educ.

[20] Paranjape K, Schinkel M, Nannan Panday R, Car J, Nanayakkara P (2019). Introducing artificial intelligence training in medical education. JMIR Med Educ.

[21] Long D, Magerko B (2020). What is AI Literacy? Competencies and Design Considerations. Proceedings of the 2020 CHI conference on human factors in computing systems. ACM.

[22] Machleid F, Kaczmarczyk R, Johann D, Balčiūnas J, Atienza-Carbonell B, von Maltzahn F (2020). Perceptions of digital health education among European medical students: mixed methods survey. J Med Internet Res.

[23] Poncette AS, Glauert DL, Mosch L, Braune K, Balzer F, Back DA (2020). Undergraduate medical competencies in digital health and curricular module development: mixed methods study. J Med Internet Res.

[24] Ahuja AS (2019). The impact of artificial intelligence in medicine on the future role of the physician. PeerJ..

[25] Wartman SA, Combs CD (2018). Medical education must move from the information age to the age of artificial intelligence. Acad Med.

[26] Pinto dos Santos D, Giese D, Brodehl S, Chon SH, Staab W, Kleinert R (2019). Medical students’ attitude towards artificial intelligence: a multicentre survey. Eur Radiol.

[27] Long D, Blunt T, Magerko B. Co-Designing AI Literacy Exhibits for Informal Learning Spaces. In: Proceedings of the ACM on Human-Computer Interaction. 2021;5(CSCW2):1–35. doi:10.1145/3476034

[28] Bishop J, Verleger M. The Flipped Classroom: A Survey of the Research. In: 2013 ASEE Annual Conference & Exposition Proceedings. ASEE Conferences; 23.1200.1–23.1200.18. doi:10.18260/1–2—22585

[29] Johnson GB (2013). Student perceptions of the flipped classroom [master thesis].

[30] Tomesko J, Cohen D, Bridenbaugh J (2022). Using a virtual flipped classroom model to promote critical thinking in online graduate courses in the United States: a case presentation. J Educ Eval Health Prof.

[31] Akçayır G, Akçayır M (2018). The flipped classroom: a review of its advantages and challenges. Comp Educ.

[32] Karaca O, Çalışkan SA, Demir K (2021). Medical artificial intelligence readiness scale for medical students (MAIRS-MS) – development, validity and reliability study. BMC Med Educ.

[33] Schiekirka S, Reinhardt D, Beibarth T, Anders S, Pukrop T, Raupach T (2013). Estimating learning outcomes from pre- and posttest student self-assessments. Acad Med.

[34] Döring N, Bortz J (2016). Forschungsmethoden Und Evaluation.

[35] Wiljer D, Salhia M, Dolatabadi E, Dhalla A, Gillan C, Al-Mouaswas D (2021). Accelerating the appropriate adoption of artificial intelligence in health care: protocol for a multistepped approach. JMIR Res Protoc.

[36] Wood EA, Ange BL, Miller DD. Are we ready to integrate artificial intelligence literacy into medical school curriculum: students and faculty survey. J Med Ed Curr Dev. 2021;8. 10.1177/23821205211024078. 10.1177/23821205211024078 PMC8239949 34250242

