# Supplementary material for: Effect of a flipped classroom course to foster medical students’ AI literacy with a focus on medical imaging: a single group pre-and post-test study
Source: BMC Med Educ. 2022 Nov 18;22:803. doi: 10.1186/s12909-022-03866-x (PMC9672614; doi:10.1186/s12909-022-03866-x)
Supplement: Supplementary file 1 — Additional file 1: Supplementary Table 1. Course curriculum, subdivided into an introduction to AI, three main modules, and the final assignment. [file 12909_2022_3866_MOESM1_ESM.docx]

Supplementary table 1. Course curriculum, subdivided into an introduction to AI, three main modules, and the final assignment.

| **Week** | **Module** |  |  |  | **Format** | **Content** |
| --- | --- | --- | --- | --- | --- | --- |
| 1 | 0  Introduction |  |  |  | Self-study  unit | Explanation of terms Artificial Intelligence. Central concepts like Machine Learning, Deep Learning, etc. are illustrated. |
| 2 | 1  Radiology |  |  |  | Self-study  unit | Illustration of various imaging techniques. Quality assurance, problems with diagnosis, etc. are reflected. |
| 3 | 1  Radiology |  |  |  | Self-study unit | Application areas of AI in radiological practice. Current research and future applications of AI in radiology. Answering the question whether human radiologists remain relevant. |
| 4 | 1  Radiology |  |  |  | Classroom lesson | Practical exercises on the use of a browser-based DICOM viewer and application-oriented information on artificial intelligence in radiology. |
| 5 | 2  Ophthalmology |  |  |  | Self-study unit | Presentation of imaging techniques in ophthalmology. Information on the (human) diagnosis of two common diseases of the eye with the help of imaging techniques. |
| 6 | 2  Ophthalmology |  |  |  | Self-study unit | Illustration of the use of AI in ophthalmological diagnosis. Comparison of advantages and disadvantages of different AI methods. Reflection on the opportunities and risks of the use of AI in ophthalmology. |
| 7 | 2  Ophthalmology |  |  |  | Classroom lesson | Beginner-oriented discussion of AI algorithms in Python using an example from ophthalmology. Comparison of diagnostic skills of the algorithm with participants competencies. |
| 8 | 3  Neuroradiology |  |  |  | Self-study unit | Most commonly used imaging modalities in neuroradiology. Exemplary findings that can be found with the respective modalities. |
| 9 | 3  Neuroradiology |  |  |  | Self-study unit | Use of AI in commercially available neuroradiology programs. Critical evaluation of the benefits of AI in neuroradiology. |
| 10 | 3  Neuroradiology |  |  |  | Classroom lesson | Practical exercises in small groups and application-oriented  information on artificial intelligence in neuroradiology. |
| 11 | Preparation of final assignm. |  |  |  | Classroom lesson | Preparation of the final assignment with appropriate information about content, scope, useful literature, etc. Evaluation of the course via an online evaluation tool. |
| 12 | Final assignment |  |  |  | Classroom lesson | Final assignment: Creating an informative interview-video similar to the videos on the MOOC-platform, in which participants reflect on AI-topics which had not been discussed throughout the course. |
