# Supplementary material for: Effect of a flipped classroom course to foster medical students’ AI literacy with a focus on medical imaging: a single group pre-and post-test study
Source: BMC Med Educ. 2022 Nov 18;22:803. doi: 10.1186/s12909-022-03866-x (PMC9672614; doi:10.1186/s12909-022-03866-x)
Supplement: Supplementary file 3 — Additional file 3. Adapted Version of MAIRS-MS Questionnaire (English version). [file 12909_2022_3866_MOESM3_ESM.pdf]

Adapted Version of MAIRS-MS Questionnaire (English version)

The questionnaire presented here is an adapted version of the questionnaire initially developed by Karaca et al. (2021)[1] The main adaptation lies in the creation of "then-" (assessment before attending the course) and "post-" (assessment after attending the course) items.

[1] Karaca O, Çalışkan SA, Demir K. Medical artificial intelligence readiness scale for medical students (MAIRS-MS) – development, validity and reliability study. BMC Med Educ. 2021;21(1):112. doi:10.1186/s12909-021-02546-6

The following 22 items survey your self-assessment of your "AI readiness", i.e. how well prepared you feel for the application and use of AI in your (future) professional environment. This involves knowledge and skills as well as your personal attitude towards AI in medicine.

The items are divided into two parts: First, please assess how competent you feel in the described area at this moment (i.e., after completing the "KI-LAURA" course). Subsequently, you are asked to recall how you would rate your competence in the described area prior to beginning the "KI-LAURA" course.

As mentioned earlier, the items are self-assessments. Accordingly, there are no right or wrong answers, and only your personal opinion matters.

Please provide your self-assessment of the following statements (in bold):

**1. I can define the basic concepts of data science.**	Not true at all	Rather not true	Somewhat true	Rather true	Completely true
at this moment (after the "KI-LAURA" course)	O	O	O	O	O
before starting the KI-LAURA course	O	O	O	O	O

**2. I can define the basic concepts of statistics.**	Not true at all	Rather not true	Somewhat true	Rather true	Completely true
at this moment (after the "KI-LAURA" course)	O	O	O	O	O
before starting the KI-LAURA course	O	O	O	O	O

**3. I can explain how AI systems are trained.**	Not true at all	Rather not true	Somewhat true	Rather true	Completely true
at this moment (after the "KI-LAURA" course)	O	O	O	O	O
before starting the KI-LAURA course	O	O	O	O	O

**4. I can define the basic concepts and terminology of AI.**	Not true at all	Rather not true	Somewhat true	Rather true	Completely true
at this moment (after the "KI-LAURA" course)	O	O	O	O	O
before starting the KI-LAURA course	O	O	O	O	O

**5. I can properly analyze the data obtained by AI in healthcare.**	Not true at all	Rather not true	Somewhat true	Rather true	Completely true

at this moment (after the “KI-LAURA” course)	O	O	O	O	O
before starting the KI-LAURA course	O	O	O	O	O

**6. I can differentiate between the functions and features of AI related tools and applications.**	Not true at all	Rather not true	Somewhat true	Rather true	Completely true
at this moment (after the “KI-LAURA” course)	O	O	O	O	O
before starting the KI-LAURA course	O	O	O	O	O

**7. I can organize workflows in accordance with the logic of AI.**	Not true at all	Rather not true	Somewhat true	Rather true	Completely true
at this moment (after the “KI-LAURA” course)	O	O	O	O	O
before starting the KI-LAURA course	O	O	O	O	O

**8. I can express the importance of data collection, analysis, evaluation and safety; for the development of AI in healthcare.**	Not true at all	Rather not true	Somewhat true	Rather true	Completely true
at this moment (after the “KI-LAURA” course)	O	O	O	O	O
before starting the KI-LAURA course	O	O	O	O	O

**9. I can use AI-based information in combination with my professional knowledge.**	Not true at all	Rather not true	Somewhat true	Rather true	Completely true
at this moment (after the “KI-LAURA” course)	O	O	O	O	O
before starting the KI-LAURA course	O	O	O	O	O

**10. I can use AI technologies effectively and efficiently in healthcare delivery.**	Not true at all	Rather not true	Somewhat true	Rather true	Completely true
at this moment (after the “KI-LAURA” course)	O	O	O	O	O
before starting the KI-LAURA course	O	O	O	O	O

**11. I can use artificial intelligence applications in accordance with its purpose.**	Not true at all	Rather not true	Somewhat true	Rather true	Completely true
at this moment (after the “KI-LAURA” course)	O	O	O	O	O
before starting the KI-LAURA course	O	O	O	O	O

**12. I can access, evaluate, use, share and create new knowledge using information and communication technologies.**	Not true at all	Rather not true	Somewhat true	Rather true	Completely true
at this moment (after the “KI-LAURA” course)	O	O	O	O	O
before starting the KI-LAURA course	O	O	O	O	O

**13. I can explain how AI applications in healthcare offer a solution to which problem.**	Not true at all	Rather not true	Somewhat true	Rather true	Completely true
at this moment (after the “KI-LAURA” course)	O	O	O	O	O
before starting the KI-LAURA course	O	O	O	O	O

**14. I find it valuable to use AI for education, service and research purposes.**	Not true at all	Rather not true	Somewhat true	Rather true	Completely true
at this moment (after the “KI-LAURA” course)	O	O	O	O	O
before starting the KI-LAURA course	O	O	O	O	O

**15. I can explain the AI applications used in healthcare services to the patient.**	Not true at all	Rather not true	Somewhat true	Rather true	Completely true
at this moment (after the “KI-LAURA” course)	O	O	O	O	O
before starting the KI-LAURA course	O	O	O	O	O

**16. I can choose the proper AI application for the problem encountered in healthcare.**	Not true at all	Rather not true	Somewhat true	Rather true	Completely true
at this moment (after the “KI-LAURA” course)	O	O	O	O	O
before starting the KI-LAURA course	O	O	O	O	O

**17. I can explain the limitations of AI technology.**	Not true at all	Rather not true	Somewhat true	Rather true	Completely true
at this moment (after the “KI-LAURA” course)	O	O	O	O	O
before starting the KI-LAURA course	O	O	O	O	O

**18. I can explain the strengths and weaknesses of AI technology.**	Not true at all	Rather not true	Somewhat true	Rather true	Completely true
at this moment (after the “KI-LAURA” course)	O	O	O	O	O
before starting the KI-LAURA course	O	O	O	O	O

**19. I can foresee the opportunities and threats that AI technology can create.**	Not true at all	Rather not true	Somewhat true	Rather true	Completely true
at this moment (after the “KI-LAURA” course)	O	O	O	O	O
before starting the KI-LAURA course	O	O	O	O	O

**20. I can use health data in accordance with legal and ethical norms.**	Not true at all	Rather not true	Somewhat true	Rather true	Completely true
at this moment (after the “KI-LAURA” course)	O	O	O	O	O
before starting the KI-LAURA course	O	O	O	O	O

**21. I can act in accordance with ethical principles while using AI technologies.**	Not true at all	Rather not true	Somewhat true	Rather true	Completely true
at this moment (after the “KI-LAURA” course)	O	O	O	O	O
before starting the KI-LAURA course	O	O	O	O	O

**22. I can follow the legal regulations regarding the use of AI technologies in healthcare.**	Not true at all	Rather not true	Somewhat true	Rather true	Completely true
at this moment (after the “KI-LAURA” course)	O	O	O	O	O
before starting the KI-LAURA course	O	O	O	O	O
