# Supplementary material for: Effect of a flipped classroom course to foster medical students’ AI literacy with a focus on medical imaging: a single group pre-and post-test study
Source: BMC Med Educ. 2022 Nov 18;22:803. doi: 10.1186/s12909-022-03866-x (PMC9672614; doi:10.1186/s12909-022-03866-x)
Supplement: Supplementary file 4 — Additional file 4. Adapted Version of MAIRS-MS Questionnaire (German version). [file 12909_2022_3866_MOESM4_ESM.pdf]

Adapted Version of MAIRS-MS Questionnaire (German version)

The questionnaire presented here is the German adapted version of the questionnaire initially developed by Karaca et al. (2021)[1] The main adaptations are the translation of the questionnaire to the German language and the creation of "then-" (assessment before attending the course) and "post-" (assessment after attending the course) items.

[1] Karaca O, Çalışkan SA, Demir K. Medical artificial intelligence readiness scale for medical students (MAIRS-MS) – development, validity and reliability study. BMC Med Educ. 2021;21(1):112. doi:10.1186/s12909-021-02546-6

Die folgenden 22 Items erheben die Selbsteinschätzung über deine "AI-Readiness", also wie gut du dich auf den Einsatz und die Nutzung von KI in deinem (zukünftigen) beruflichen Umfeld vorbereitet fühlst. Dabei geht es sowohl um Wissen und Fertigkeiten als auch um deine persönlichen Einstellungen zum Thema KI in der Medizin.

Die Items sind jeweils zweigeteilt: Erst sollst du bitte einschätzen, wie kompetent du dich im beschriebenen Bereich zum jetzigen Zeitpunkt (also nach Abschluss des KI-LAURA Kurses) fühlst. Danach sollst du dich bitte daran zurückerinnern, wie du deine Kompetenz im beschriebenen Bereich vor Beginn des KI-LAURA Kurses einschätzen würdest.

Bei den Items handelt es sich wie gesagt um eine Selbsteinschätzung. Dementsprechend gibt es keine richtigen oder falschen Antworten, sondern es geht um deine persönliche Meinung.

Bitte gib deine Selbsteinschätzung zu folgenden Aussagen (fettgedruckt) an:

**1. Ich kann die grundlegenden Konzepte von Data Science definieren.**	Trifft gar nicht zu	Trifft weniger zu	Trifft teil-weise zu	Trifft ziemlich zu	Trifft völlig zu
zum jetzigen Zeitpunkt (nach dem KI-LAURA Kurs)	O	O	O	O	O
vor Beginn des KI-LAURA Kurses	O	O	O	O	O

**2. Ich kann die grundlegenden Konzepte von Statistik definieren.**	Trifft gar nicht zu	Trifft weniger zu	Trifft teil-weise zu	Trifft ziemlich zu	Trifft völlig zu
zum jetzigen Zeitpunkt (nach dem KI-LAURA Kurs)	O	O	O	O	O
vor Beginn des KI-LAURA Kurses	O	O	O	O	O

**3. Ich kann erklären, wie KI-Systeme trainiert werden.**	Trifft gar nicht zu	Trifft weniger zu	Trifft teil-weise zu	Trifft ziemlich zu	Trifft völlig zu
zum jetzigen Zeitpunkt (nach dem KI-LAURA Kurs)	O	O	O	O	O
vor Beginn des KI-LAURA Kurses	O	O	O	O	O

**4. Ich kann die grundlegenden Konzepte und die Terminologie von KI definieren.**	Trifft gar nicht zu	Trifft weniger zu	Trifft teil-weise zu	Trifft ziemlich zu	Trifft völlig zu
zum jetzigen Zeitpunkt (nach dem KI-LAURA Kurs)	O	O	O	O	O
vor Beginn des KI-LAURA Kurses	O	O	O	O	O

**5. Ich kann die von KI im Gesundheitswesen gewonnenen Daten richtig analysieren.**	Trifft gar nicht zu	Trifft weniger zu	Trifft teil-weise zu	Trifft ziemlich zu	Trifft völlig zu
zum jetzigen Zeitpunkt (nach dem KI-LAURA Kurs)	O	O	O	O	O
vor Beginn des KI-LAURA Kurses	O	O	O	O	O

**6. Ich kann zwischen den Funktionen und Merkmalen von KI-bezogenen Tools und Anwendungen differenzieren.**	Trifft gar nicht zu	Trifft weniger zu	Trifft teil-weise zu	Trifft ziemlich zu	Trifft völlig zu
zum jetzigen Zeitpunkt (nach dem KI-LAURA Kurs)	O	O	O	O	O
vor Beginn des KI-LAURA Kurses	O	O	O	O	O

**7. Ich kann Arbeitsabläufe nach der Logik der KI organisieren.**	Trifft gar nicht zu	Trifft weniger zu	Trifft teil-weise zu	Trifft ziemlich zu	Trifft völlig zu
zum jetzigen Zeitpunkt (nach dem KI-LAURA Kurs)	O	O	O	O	O
vor Beginn des KI-LAURA Kurses	O	O	O	O	O

**8. Ich kann die Bedeutung von Datenerfassung, -analyse, -auswertung und -sicherheit für die Entwicklung von KI im Gesundheitswesen darstellen.**	Trifft gar nicht zu	Trifft weniger zu	Trifft teil-weise zu	Trifft ziemlich zu	Trifft völlig zu
zum jetzigen Zeitpunkt (nach dem KI-LAURA Kurs)	O	O	O	O	O
vor Beginn des KI-LAURA Kurses	O	O	O	O	O

**9. Ich kann KI-basierte Informationen in Kombination mit meinem fachlichen Wissen nutzen.**	Trifft gar nicht zu	Trifft weniger zu	Trifft teil-weise zu	Trifft ziemlich zu	Trifft völlig zu
zum jetzigen Zeitpunkt (nach dem KI-LAURA Kurs)	O	O	O	O	O
vor Beginn des KI-LAURA Kurses	O	O	O	O	O

**10. Ich kann KI-Technologien effektiv und effizient in der Gesundheitsversorgung einsetzen.**	Trifft gar nicht zu	Trifft weniger zu	Trifft teil-weise zu	Trifft ziemlich zu	Trifft völlig zu
zum jetzigen Zeitpunkt (nach dem KI-LAURA Kurs)	O	O	O	O	O
vor Beginn des KI-LAURA Kurses	O	O	O	O	O

**11. Ich kann KI-Anwendungen ihrem Zweck entsprechend einsetzen.**	Trifft gar nicht zu	Trifft weniger zu	Trifft teil-weise zu	Trifft ziemlich zu	Trifft völlig zu
zum jetzigen Zeitpunkt (nach dem KI-LAURA Kurs)	O	O	O	O	O
vor Beginn des KI-LAURA Kurses	O	O	O	O	O

**12. Ich kann mit Informations- und Kommunikationstechnologien auf Wissen zugreifen, es bewerten, nutzen, teilen und neues Wissen schaffen.**	Trifft gar nicht zu	Trifft weniger zu	Trifft teil-weise zu	Trifft ziemlich zu	Trifft völlig zu
zum jetzigen Zeitpunkt (nach dem KI-LAURA Kurs)	O	O	O	O	O
vor Beginn des KI-LAURA Kurses	O	O	O	O	O

**13. Ich kann erklären, welche KI-Anwendungen im Gesundheitswesen eine Lösung für welches Problem bieten.**	Trifft gar nicht zu	Trifft weniger zu	Trifft teil-weise zu	Trifft ziemlich zu	Trifft völlig zu
zum jetzigen Zeitpunkt (nach dem KI-LAURA Kurs)	O	O	O	O	O
vor Beginn des KI-LAURA Kurses	O	O	O	O	O

**14. Ich finde es wertvoll, KI für Bildungs-, Dienstleistungs- und Forschungszwecke einzusetzen.**	Trifft gar nicht zu	Trifft weniger zu	Trifft teil-weise zu	Trifft ziemlich zu	Trifft völlig zu
zum jetzigen Zeitpunkt (nach dem KI-LAURA Kurs)	O	O	O	O	O
vor Beginn des KI-LAURA Kurses	O	O	O	O	O

**15. Ich kann dem Patienten/der Patientin die im Gesundheitswesen eingesetzten KI-Anwendungen erklären.**	Trifft gar nicht zu	Trifft weniger zu	Trifft teil-weise zu	Trifft ziemlich zu	Trifft völlig zu
zum jetzigen Zeitpunkt (nach dem KI-LAURA Kurs)	O	O	O	O	O
vor Beginn des KI-LAURA Kurses	O	O	O	O	O

**16. Ich kann die richtige KI-Anwendung für das im Gesundheitswesen auftretende Problem auswählen.**	Trifft gar nicht zu	Trifft weniger zu	Trifft teil-weise zu	Trifft ziemlich zu	Trifft völlig zu
zum jetzigen Zeitpunkt (nach dem KI-LAURA Kurs)	O	O	O	O	O
vor Beginn des KI-LAURA Kurses	O	O	O	O	O

**17. Ich kann die Grenzen von KI-Technologie erklären.**	Trifft gar nicht zu	Trifft weniger zu	Trifft teil-weise zu	Trifft ziemlich zu	Trifft völlig zu
zum jetzigen Zeitpunkt (nach dem KI-LAURA Kurs)	O	O	O	O	O
vor Beginn des KI-LAURA Kurses	O	O	O	O	O

**18. Ich kann die Stärken und Schwächen von KI-Technologie erklären.**	Trifft gar nicht zu	Trifft weniger zu	Trifft teil-weise zu	Trifft ziemlich zu	Trifft völlig zu
zum jetzigen Zeitpunkt (nach dem KI-LAURA Kurs)	O	O	O	O	O
vor Beginn des KI-LAURA Kurses	O	O	O	O	O

**19. Ich kann die Chancen und Gefahren, die KI-Technologie mit sich bringen kann, voraussehen.**	Trifft gar nicht zu	Trifft weniger zu	Trifft teil-weise zu	Trifft ziemlich zu	Trifft völlig zu
zum jetzigen Zeitpunkt (nach dem KI-LAURA Kurs)	O	O	O	O	O
vor Beginn des KI-LAURA Kurses	O	O	O	O	O

**20. Ich kann Gesundheitsdaten im Einklang mit rechtlichen und ethischen Normen nutzen.**	Trifft gar nicht zu	Trifft weniger zu	Trifft teil-weise zu	Trifft ziemlich zu	Trifft völlig zu
zum jetzigen Zeitpunkt (nach dem KI-LAURA Kurs)	O	O	O	O	O
vor Beginn des KI-LAURA Kurses	O	O	O	O	O

**21. Ich kann bei der Nutzung von KI-Technologien im Einklang mit ethischen Prinzipien handeln.**	Trifft gar nicht zu	Trifft weniger zu	Trifft teil-weise zu	Trifft ziemlich zu	Trifft völlig zu
zum jetzigen Zeitpunkt (nach dem KI-LAURA Kurs)	O	O	O	O	O
vor Beginn des KI-LAURA Kurses	O	O	O	O	O

**22. Ich kann die gesetzlichen Bestimmungen für den Einsatz von KI-Technologien im Gesundheitswesen einhalten.**	Trifft gar nicht zu	Trifft weniger zu	Trifft teil-weise zu	Trifft ziemlich zu	Trifft völlig zu
zum jetzigen Zeitpunkt (nach dem KI-LAURA Kurs)	O	O	O	O	O
vor Beginn des KI-LAURA Kurses	O	O	O	O	O
